# Potential Role of Specific Antibodies as Important Vaccine Induced Protective Mechanism against *Aeromonas salmonicida* in Rainbow Trout

**DOI:** 10.1371/journal.pone.0046733

**Published:** 2012-10-08

**Authors:** Kasper Rømer Villumsen, Inger Dalsgaard, Lars Holten-Andersen, Martin Kristian Raida

**Affiliations:** 1 Laboratory of Aquatic Pathobiology, Faculty of Health and Medical Sciences, University of Copenhagen, Denmark; 2 National Veterinary Institute, Technical University of Denmark, Frederiksberg, Denmark; Auburn University, United States of America

## Abstract

Furunculosis caused by infection with *Aeromonas salmonicida* subsp. *salmonicida* has been a known threat to aquaculture for more than a century. Efficient prophylactic approaches against this disease are essential for continued growth of salmonid aquaculture. Since the introduction of successful oil-adjuvanted vaccines in the early 1990's, a number of studies have been published on the protective as well as adverse effects of these vaccines. Most studies focus on vaccination of salmon (*Salmo salar*). However, rainbow trout (*Oncorhynchus mykiss*) are also very susceptible to infection and are vaccinated accordingly. In this study we have examined the protection against infection with a Danish strain of *A. salmonicida* in both vaccinated and non-vaccinated rainbow trout. A commercial and an experimental auto-vaccine were tested. The protective effects of the vaccines were evaluated through an *A. salmonicida* challenge 18 weeks post vaccination. Both vaccines resulted in a significantly increased survival in the vaccinated fish during a 28 day challenge period relative to non-vaccinated fish (P = 0.01 and P = 0.001 for the commercial and experimental vaccine, respectively). Throughout the entire experiment, the presence of specific antibodies in plasma was monitored using ELISA. A significant increase in specific antibody levels was seen in fish vaccinated with both vaccines during the 18 weeks between vaccination and challenge. Within 3 days post challenge, a significant decrease in specific antibodies occurred in vaccinated fish. A positive correlation was found between mean levels of specific antibodies pre challenge and overall survival. This correlation, along with the observed depletion of antibodies during the initial phase of infection, suggests that specific antibodies play an essential role in vaccine mediated protection against *A. salmonicida* in rainbow trout.

## Introduction

*Aeromonas salmonicida* subsp. *salmonicida* (hereafter referred to as *A. salmonicida*) has been known as the causative agent of furunculosis since the first cases were described in the late 19^th^ century [1]. With the emergence of modern aquaculture, this Gram-negative bacterium has developed into one of the most significant bacterial infections in cultured fish across the world, affecting a wide range of host species [2]. This has resulted in substantial economic losses, particularly in the production of salmonid species. In order to control the disease, great efforts have been put into developing efficient prophylactic tools to prevent outbreaks in aquaculture. Initial studies on oral vaccination of cutthroat trout proved successful after periods of continuous immunization [3]. Immersion has also been proven to provide protection against mortalities in Chinook salmon, rainbow trout and Atlantic salmon [4]–[6]. However, other experiments in Atlantic salmon have since demonstrated limited long-term effects of both oral and immersion vaccination when compared to intraperitoneal (i.p.) injection of adjuvanted vaccines [7]. Such short term protection will not meet the needs of modern aquaculture, since reduced long-term protection will result in an increased need for antibiotics during disease outbreaks, and an increase in disease related mortalities.

Several studies have investigated the protection provided by adjuvanted i.p. injected vaccines against *A. salmonicida*. Early on, studies using injected vaccines in brook trout and brown trout reported long-lasting, effective protection when using a mineral oil adjuvant [8]. Later studies performed in Atlantic salmon have shown similar results. Using oil-adjuvanted vaccines, a long-term, high level of protection was seen for up to at least 6 months post vaccination, along with significantly elevated antibody levels against whole-cell bacteria and membrane proteins [9]–[11].

Studies have also been done on the antibody response of rainbow trout towards various preparations of extracellular products [12], [13]. However, these studies conclude that the response is limited in effect. Neither of these two studies includes survival data.

The oil-adjuvanted bacterin based vaccine has now become the main one for vaccinations of salmonids against *A. salmonicida* in commercial aquaculture [14]–[16]. While providing long-lasting, superior levels of protection, oil-adjuvanted vaccines have also been shown to be associated with adverse effects, ranging from local observations such as pigmentation of tissues and intra-abdominal adhesions [10], [17], [18] to systemic autoimmunity and pathological changes in numerous tissues [19], [20]. As with many other studies, these have focused on salmon. Relative to salmon, few vaccination studies have been performed on rainbow trout. Recently, studies have shown that rainbow trout are more susceptible to *A. salmonicida* than salmon [21]. A survey of freshwater aquaculture have shown that few clinical infections occur in freshwater [22]. It has been suggested that the trout are infected in freshwater farms, carrying the infection to seawater, where stress and high temperature may result in an outbreak of *A. salmonicida* with resulting high mortality rates [15]. Vaccinations are usually given prior to their transfer to sea cages, and reduce mortalities in the vaccinated fish during infections [15], [23].

The aim of this study was to look into protective effects of vaccination of rainbow trout against *A. salmonicida*,focusing on potential humoral effects. This was done by investigating the level of protection induced against an experimental bacterial infection by two different vaccines. The induction of specific antibody production was examined post vaccination, and during the bacterial challenge. Finally, a potential correlation between antibody titer and survival during bacterial challenge was tested. This should provide insight into potentially protective effects of vaccine induced specific antibodies during an infection with *A. salmonicida*.

## Materials and Methods

### Fish

1500 rainbow trout were hatched and reared under pathogen-free conditions at the Bornholm Salmon Hatchery (Nexø, Denmark). The pathogen-free status was achieved by introducing certified disinfected eggs into a recirculated system at the hatchery. From the time of vaccination until time of challenge the fish were kept at the hatchery. Here the fish were kept in 500 L, recirculated tanks at 14±1°C with a water flow of 25 L/min, and a photoperiod of 7 hours per day. The fish were fed 1% of body weight daily. Before bacterial challenge, the fish were transferred to the experimental facilities at the University of Copenhagen in Frederiksberg, Denmark. Before initiating the challenge, the pathogen-free status was confirmed by standard bacteriological examination methods in the laboratory. The Committee for Animal Experimentation, Ministry of Justice, Denmark, approved this study under license nr. 2012/561-147. The study was conducted according to the ethical guidelines stated in the license.

### Experimental auto vaccine

A Danish strain of *Aeromonas salmonicida* subsp. *salmonicida* (040617-1/1A, challenge strain) was grown in heart infusion broth (infusion made from 50% v/w beef heart, 1% v/w Bacto Tryptose, 0.5% v/w NaCl, pH 7.4) for 48 h at a constant temperature of 20°C with continuous shaking. The number of colony forming units (CFU) per ml was estimated by triplicate plating of a ten-fold dilution series of the bacterial culture. The culture was then inactivated by addition of formalin to a final volume of 2%, after which the inactivation was confirmed by a series of subsequent plating on blood agar plates which yielded no CFU. After washing in phosphate buffered saline (PBS), the bacterin was adjusted to 4×10^9^ CFU/ml in PBS. Immediately prior to administration, the bacterin was thoroughly mixed 1∶1 with Freund's incomplete adjuvant (Sigma-Aldrich- F5506). An injection dose of a total of 50 µl experimental vaccine per fish therefore contained 1×10^8^ CFU.

### Vaccinations

Before vaccination, the fish (9.3±0.8 grams) were anaesthetized by immersion in Benzoak VET (ACD Pharmaceuticals AS) (56 mg/L, aerated water). Fish were vaccinated and grouped as follows: I) 300 fish were kept as unhandled controls, II) 300 fish were vaccinated intraperitoneally (i.p.) with 50 µl of commercial furunculosis vaccine (AlphaJect® 3000, PHARMAQ AS, Overhalla, Norway), III) 300 fish were injected i.p. with 50 µl AlphaJect® adjuvant (PHARMAQ AS, Overhalla, Norway), IV) 300 fish were injected i.p. with 50 µl experimental vaccine, V) 300 fish were injected i.p. with 50 µl Freund's incomplete adjuvant in PBS (1∶1).

The commercially available AlphaJect 3000 vaccine is a trivalent vaccine containing a formaline-inactivated strain of *A. salmonicida* subsp. *salmonicida* as well as a strain of both *Vibrio anguillarum* serotype O1 and O2a, mixed with a liquid paraffin adjuvant.

The fish were kept for 129 days at 14°C post vaccination, a total of 1806 degree days, before challenge.

### Sampling

On the day of vaccination, blood samples were taken from 10 unhandled, unvaccinated fish. Subsequently, blood samples were taken 3, 10 and 18 weeks post vaccination, as well as 1, 3 and 28 days post infection from 5 fish from each experimental group. Fish were euthanized in an overdose of MS-222 (200 mg/L) (Sigma-Aldrich Inc.), after which blood was drawn from the *Vena caudalis* using a 25G needle, and a heparinized syringe. The sample was then centrifuged at 4000G for 10 min. The plasma fraction was transferred to separate tubes, and kept at −80°C until they were processed for ELISA measurement of antibody content.

### Bacterial challenge

Fish were transported from the hatchery to the experimental infection facilities at the University of Copenhagen, and upon arrival each experimental group was transferred into duplicate 200 L tanks each holding 30 fish, one tank for monitoring mortality and one holding 30 fish for sampling during the challenge experiment. The fish were left to acclimatize for 18 days at a constant room temperature of 14°C before the challenge was started. The fish were fed to satiation daily. Immediately prior to challenge, the average weight of the fish was 32.6±1.6 grams (n = 30).

A challenge dose of the *A. salmonicida* challenge isolate was grown as described above, resulting in 1.2×10^8^ CFU/ml. A bath challenge was performed by transferring fish from each tank to separate tanks containing a 3 L, 1∶20 dilution of the bacterial suspension (6×10^6^ CFU/ml). The exposure period was 1 hour, during which a constant flow of air was provided and the fish were monitored closely. After challenge the fish were transferred to their original tanks, and kept here for 28 days. The fish were monitored several times a day. Fish displaying a clear lack of appetite, isolated behavior, dark coloration and difficulties maintaining equilibrium were regarded as moribund and euthanized (200 mg MS-222/l).

### Enzyme-Linked Immunosorbent Assay (ELISA)

ELISA was performed with all collected plasma as described by Raida, Nylen & Holten-Andersen [24]. Briefly, wells on microtiter plates (NUNC Maxisorp, Thermo Fisher Scientific Inc.) were coated overnight at 4°C with a sonicate of the *A.salmonicida* challenge strain by adding the sonicate suspended in bicarbonate buffer (pH 9.6)(Sigma-Aldrich Inc.) at a concentration of 5 µg antigen protein/ml. Each well was then washed in 250 µl washing buffer (PBS+0.2% Tween 20), incubated for 1 hour in blocking buffer (PBS+0.1% Tween 20+2% bovine serum albumin (BSA)) and subsequently washed in 250 µl washing buffer. All washing steps consisted of 3 washes in wash buffer.

The antibody titers were assayed by serial 5-fold dilutions. All samples were processed in duplicate. Dilutions were made with assay diluent (PBS+1% BSA+1% Tween 20), and a volume of 100 µl was added to each duplicate well and incubated over night at 4°C. On each plate, 4 wells were incubated with pure diluent instead of diluted sample, for measurement of background absorbance. An additional total of 8 wells were incubated with two different internal standard plasma samples for interplate calibration.

After wash, 100 µl mouse-anti-salmonid Ig (AbD Serotec, diluted 1∶500) was added to each well, and left at room temperature for 1 h on a rotary shaker. After a subsequent washing step, 100 µl horseradish-peroxidase labeled rabbit-anti-mouse Ig (AbD Serotec, diluted 1∶500) was added, and the plate was placed on the shaker at room temperature for 1 h. Each well was finally washed again before 100 µl TMB PLUS substrate (AbD serotec) was added. The plate was placed on the rotary shaker, and monitored for peroxidase reaction for 10 minutes. The reaction was then stopped, by adding 100 µl 1 M HCl, and the plate was analyzed in an ELISA plate-reader (Epoch, BioTek Instruments Inc.) at 450 nm. During subsequent analysis, the average background absorbance for each plate was subtracted from the measured absorbance from the sample measurements of that plate. For statistical analysis, the average optical density (OD) measured from the duplicate 1∶25 dilution of each sample was used. For calculation of specific antibody titers, the average value for each dilution step was plotted for each individual in each group (y-axis) against the log_10_-transformed dilution steps (x-axis). Linear regression was performed on the descending, linear section of the resulting sigmoidal plot. The average measured background values relevant for each sample were plotted as a horizontal line, and the titer for each fish was defined as the x-coordinate for the intersection between the regression –line and the average background.

### Statistical analysis

All statistical analysis was performed using GraphPad Prism 5 (GraphPad Software, Inc.).

Mortality data were analyzed using the Kaplan-Meier method to analyze the survival curves. Groups were examined against each other using the log-rank test to test for significant differences. The relative percent survival was calculated using the following equation:
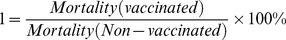
ELISA results in fig. 2 were examined using a one-way ANOVA with a Tukey post test to identify significant differences between groups. Due to non-Gaussian distribution of data, ELISA titers were analyzed using Kruskal-Wallis, with Dunn's post test for differences between between groups. Correlation analysis was performed using the Pearson method. In all statistical analysis, P-values<0.05 were considered statistically significant.

## Results

### Bacterial challenge

**Figure 1:**
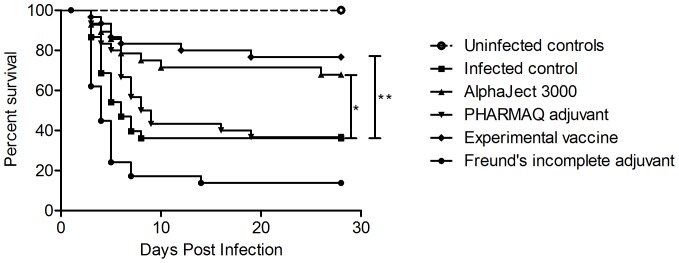
Survival curves from bacterial challenge. Survival curves representing the outcome of the bacterial challenge. N = 30 for each group, except AlphaJect 3000 (N = 28). The two vaccinated groups showed significantly higher survival than infected controls (P = 0.001 and 0.01 for experimental vaccine and AlphaJect 3000, respectively), as well as their respective adjuvants (P = 0.0001 and 0.02 for experimental vaccine and AlphaJect 3000, respectively). Asterisks indicate statistically significant difference between treated group and infected controls (* = P<0.05, ** = P<0.01).

The results from the bacterial challenge with *A. salmonicida* are shown in fig. 1. The onset of mortality was 3 days post challenge for all infected groups. Mortalities accumulated within the first 9 days post challenge, and then settled with little additional increase for the remainder of the challenge period. Both vaccines gave significant reductions in mortality compared to the non-vaccinated control group, with an RPS of 63.48% (P = 0.001) for the experimental auto vaccine and 49.69% (P = 0.01) for the commercial vaccine. Additionally, both vaccines showed significantly reduced mortalities compared to their respective adjuvant formulations (P = 0.0001 and P = 0.02 for experimental and commercial formulation, respectively). There was no significant difference between the two vaccines.

### ELISA

**Figure 2:**
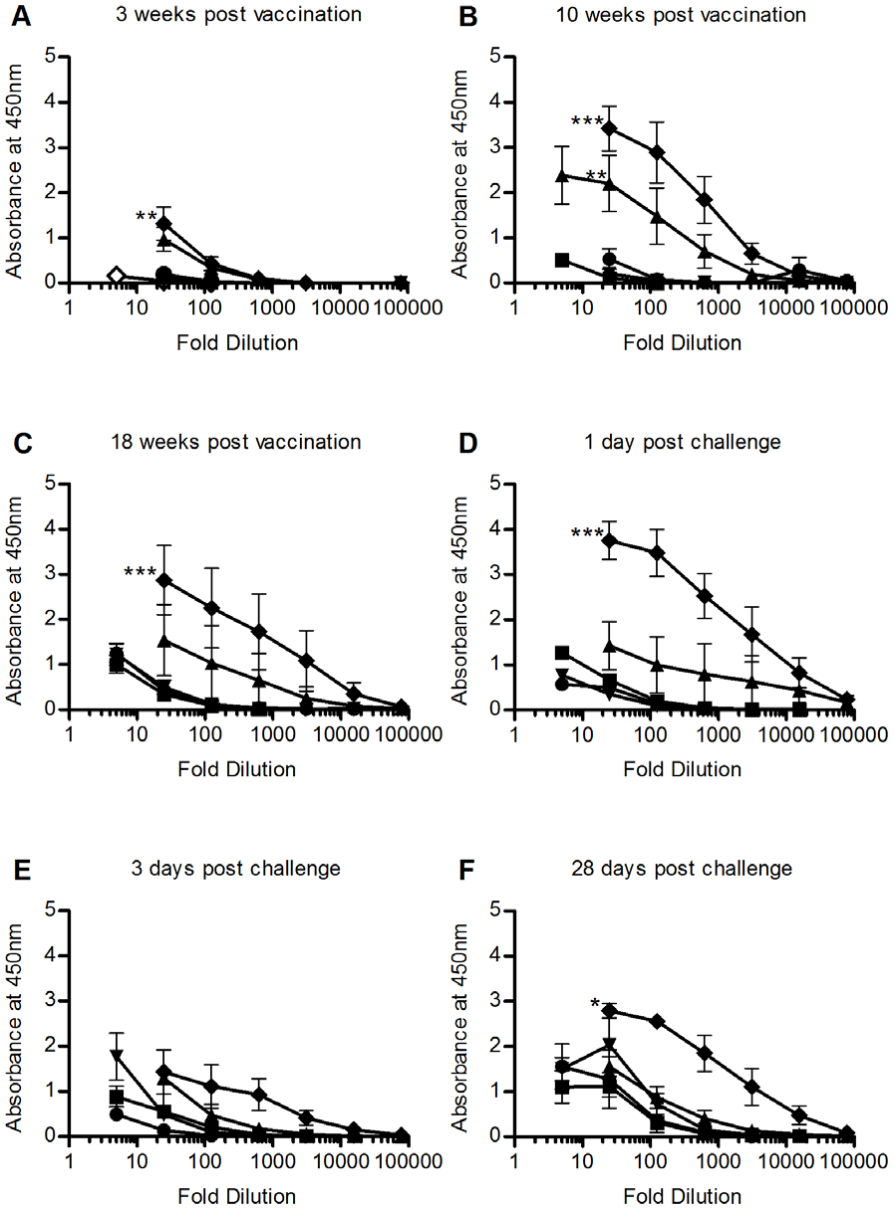
Specific antibody response. Figures 2A–F show the results of the ELISA performed on sera collected from each experimental group at each of the 7 sampling time points. ***Empty diamond:*** *Pre vaccination* ***Filled square:*** *control,* ***filled diamond:*** *experimental vaccine,* ***Filled circle:*** *Freunds incomplete adjuvant,* ***Filled upward facing triangle:*** *AlphaJect 3000,* ***Filled downward facing triangle:*** *PHARMAQ adjuvant*. ANOVA analysis of ELISA results were performed on the measured absorbance of the separate 1∶25 dilutions in each group. Asterisks indicate statistically significant difference between treated group and infected controls (* = P<0.05, ** = P<0.01, *** = P<0.001). All data shown are mean absorbance ± SEM.

**Figure 3:**
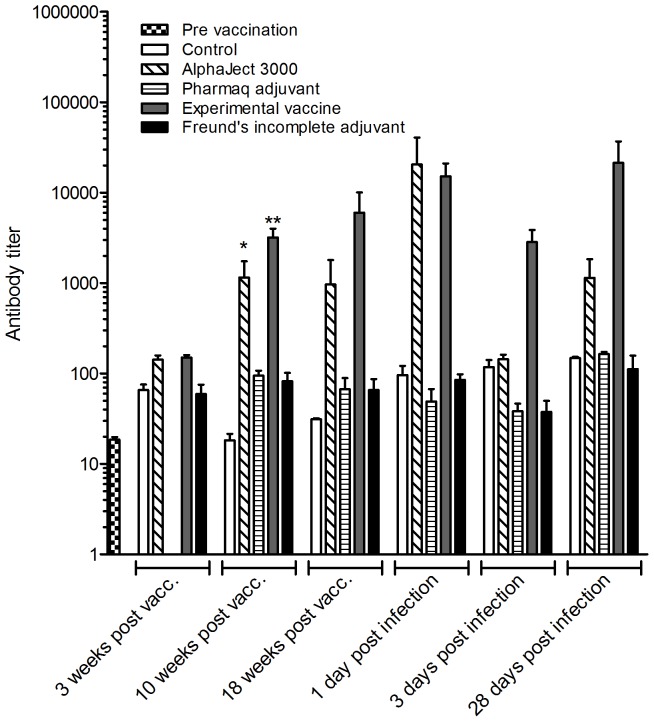
Antibody titers. Mean calculated titers ± SEM for each group at each time point. The titers were calculated as described in the main text. Differences in antibody titer between control and treated groups were analyzed using the Kruskal-Wallis method. Asterisks indicate statistically significant difference between treated group and infected controls (* = P<0.05, ** = P<0.01). See legend for identification of the experimental groups.

The levels of specific antibodies were monitored at 7 different time points: immediately prior to vaccination, 3, 10 and 18 weeks post vaccination, and 1, 3 and 28 days post challenge. The results are shown in fig. 2. At all three time points between vaccination and challenge the experimental vaccine resulted in a significantly increased antibody level compared to non-vaccinated controls, while the commercial vaccine showed significantly increased antibody levels relative to non-vaccinated controls at 10 weeks post vaccination. None of the two adjuvant injected groups gave significant increases in antibody levels compared to non-vaccinated controls. A significant drop in absorbance was seen for the experimental vaccine between day 1 and 3 post challenge. No decrease was seen in the commercial vaccine during the same period. Fig. 3 shows a comparison of the calculated antibody titers for each group at each time point. Antibody titers displayed non-Gaussian distribution within groups, and were analyzed using the non-parametric Kruskal-Wallis method. At 10 weeks post vaccination the antibody titers of both vaccines were significantly higher than that of the control group.

### Correlation between ELISA and challenge data

**Figure 4:**
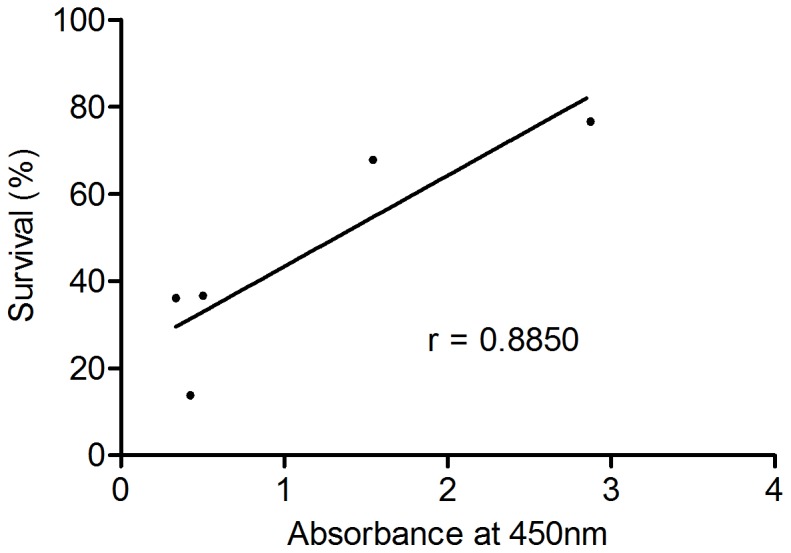
Correlation between antibodies and survival. Figure 3 shows a xy-plot of the survival of each group (N = 30, except AlphaJect 3000 (N = 28)) and the average measured absorbance in the 1∶25 dilution of individual plasma samples from fish in each group (N = 5). Plasma samples are taken 18 weeks post vaccination, immediately prior to bacterial challenge. The correlation was calculated using the Pearson method: P = 0.046, r = 0.885.

Fig. 4 shows the result of plotting the survival of each experimental group against the average antibody level measured for the respective groups. A statistically significant, positive correlation was found between increasing antibody and increasing survival (P = 0.046, r = 0.885).

## Discussion

The aims of this study were to assay the level of protection provided by the two different vaccines against infection with *A. salmonicida*, to investigate the development of specific antibody responses and to get an insight into the potential protection afforded by these antibody responses. Several studies on the effects of vaccination have been performed on salmon, possibly due to their economic role in modern aquaculture. However, some works have focused on rainbow trout, from the initial attempts on vaccination made by Duff in the 1940's [3], over later immunization studies by Krantz *et al.* [8], and on to the work of Hastings, Stapleton and Ellis on the antibody responses towards extracellular products of *A. salmonicida* [12], [13]. This study was initiated to provide a collected view of the role of antibodies in the induction of protection induced by the dominant oil adjuvanted i.p. vaccination of rainbow trout.

The results from the bacterial challenge experiment show that the vaccinated rainbow trout in this study were significantly protected relative to the non-vaccinated ones. While the graph illustrates that all groups exhibit a similar timeframe between the onset and the end of mortality, the two vaccinated groups show a low mortality within this timeframe, whereas the adjuvant groups and the infected control undergo a steep decline in survival over the same period of time. Similar patterns are seen in studies conducted on salmon both after bath [11] and co-habitation challenges [7], [10]. This could indicate that a high level of immediate protection during the earliest stages of infection is required for successful survival during infection. A possible source of protection is a competent humoral immune barrier, such as a readily available pool of specific antibodies. The significant levels of protection in this experiment are seen 4 months post vaccination. Previous studies by Midtlyng *et al.* have shown significant protection for up to 6 months in Atlantic salmon vaccinated with oil-adjuvanted vaccines against *A. salmonicida* [10].

Both vaccinated groups developed significantly increased levels of specific antibodies, able to recognize a sonicate of the bacterial challenge strain during the first 10 weeks post vaccination. The fish vaccinated with the experimental vaccine reaches significantly increased antibody levels after 3 weeks, and continues to exhibit a significant increase during the remaining 15 weeks until the bacterial challenge. The differences in antibody levels between the experimental and the commercial vaccine could, in part, be due to the fact that the bacterial strain used in the vaccine and the ELISA is identical. The results are similar to those seen in *S. salar* after vaccination with both oil- and aluminum salt-adjuvanted vaccines [9], [25]. Erdal & Reitan [9] measured the antibody levels towards whole bacteria, as well as both A-layer protein and lipopolysaccharide. In their studies antibody levels against whole bacteria were significantly elevated at 3 weeks post vaccination, and remained high for the remainder of their 8 week experimental period. Antibody levels against the more specific bacterial components display a less consistent response during the same time period. Midtlyng *et al.* also reported that salmon vaccinated with an oil-adjuvanted vaccine, showed increasing antibody titers throughout a 6 month period [10]. In the present experiment, the commercial vaccine reached a significant increase after 10 weeks. However, at 18 weeks post vaccination, the antibody levels of the commercial vaccine were no longer significantly different from that of the non-vaccinated controls. They are, however, still elevated compared to the non-vaccinated controls. Krantz *et al.* showed elevated antibody levels in brown trout after an oil adjuvanted vaccination, sustaining the increased levels for at least 24 months [8]. Bricknell *et al.* showed elevated antibody levels after un-adjuvanted vaccination of salmon, lasting at least 9 months [11]. The same study reports vaccine induced protection 9 months after vaccination, well beyond the timeline presented in this study. Another interesting observation to be made is the significant decrease in the level of antibodies seen in the fish vaccinated with the experimental vaccine between day 1 and day 3 post challenge. Bricknell *et al.* report similar findings in salmon [11]. Their study reports that the development of antibody titers against iron-regulated outer membrane proteins and polysaccharides in vaccinated salmon appears to be limited by a bacterial challenge when compared to non-challenged, vaccinated salmon. They propose several possible explanations. One of these is that the decrease in plasma antibody levels could be due to formation of antibody∶antigen complexes, reducing the number of free, circulating antibodies. The authors later suggest the role of immunosuppressing serine proteases [26]. However, depending on the turnover of circulating antibodies, taking into account the timeframe of just 2 days and the fact that the decrease occurs at the time of the onset of mortality, we believe that the decrease seen in this study could be due to a depletion of antibodies, either in formation of antigen∶antibody complexes, or opsonization of such complexes.

When comparing the antibody levels in fig. 2 with the calculated antibody titers shown in fig. 3, it is clear that both vaccines induce development of large pools of specific antibodies, indicated by the large increases in antibody titer. Comparing the survival data shown in fig. 1 with the antibody measurements shown in fig. 2, the two vaccinated groups show both the highest survival percentages, as well as the highest antibody titers. Since the plasma samples were taken immediately prior to the bacterial challenge, these antibody titers represent the available pools of specific antibodies at the time of challenge for each group. This again proves a possible connection between the survival data seen in fig. 1 and the results of the ELISA shown in fig. 2, since these pools reflect the potential protective antibody mediated defense against the pathogen.

Recently, ELISA performed on blood samples from salmon vaccinated against *A. salmonicida* was proposed as a means of conducting vaccine batch testing by Romstad, *et al* [25]. In their study, a statistically significant, positive correlation was found between increased levels of specific antibodies and increased survival in the vaccinated fish. Bricknell *et al.* also find correlations between specific antibodies and protection [11]. Our results provide basis for a similar analysis, and show a significant correlation between antibody level and survival.

The work presented here has not dealt with the possible cell-mediated immune response against *A. salmonicida*. However, we have shown a significant level of protection against infection induced by a commercial, as well as an experimental vaccine. Both vaccines induced production of specific antibodies, reaching significantly elevated levels compared to those of non-vaccinated controls within 10 weeks of vaccination. Two factors indicate a connection between the antibody production and protection induced by the vaccinations: a positive correlation between antibody level prior to challenge and survival during challenge, as well as an apparent depletion of plasma antibodies in the early, crucial phase of the infection. These two factors correlate well with the experiences from studies on vaccination of salmon mentioned earlier, and emphasize the protective role of specific antibodies against *A. salmonicida*. The exact nature of this protective effect remains undisclosed, and future studies are needed to determine the details of the protective effects, as well as potential interplay between humoral and cell-mediated immune responses. Hopefully this will pave the way for future studies on vaccination of rainbow trout against *A. salmonicida*.
