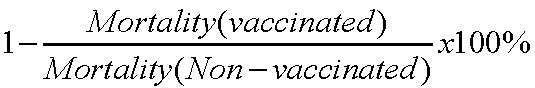# Correction: Potential Role of Specific Antibodies as Important Vaccine Induced Protective Mechanism against *Aeromonas salmonicida* in Rainbow Trout

**DOI:** 10.1371/annotation/48f605ab-75a9-4f93-9b4a-ab4358cd15d6

**Published:** 2012-11-14

**Authors:** Kasper Rømer Villumsen, Inger Dalsgaard, Lars Holten-Andersen, Martin Kristian Raida

In the Materials and Methods section, a formula for the calculation of the relative percent survival is presented incorrectly. A "-" (minus) has been replaced with an "=" (equal to). The correct equation can be viewed here: